# Effect of Tensile Stress on the Oxide Properties of a Nickel-Based Alloy 600 in Simulated PWR Secondary Water

**DOI:** 10.3390/ma14216460

**Published:** 2021-10-28

**Authors:** Byung-Joon Bae, Jeoh Han, Jongsup Hong, Do-Haeng Hur

**Affiliations:** 1 Materials Safety Technology Development Division, Korea Atomic Energy Research Institute, Daejeon 34057, Korea; qoqudwns0302@naver.com (B.-J.B.); jeohhan@kaeri.re.kr (J.H.); 2 Division of Mechanical Engineering, Yonsei University, Seoul 03722, Korea; jongsup.hong@yonsei.ac.kr

**Keywords:** tensile stress, Alloy 600, corrosion, oxide property, plastic deformation, electrochemical impedance, defect density, stress corrosion cracking, pressurized water reactor

## Abstract

The purpose of this work was to examine the effect of tensile stress on the oxide properties of a nickel-based Alloy 600 that was exposed to simulated nuclear steam generator water at 340 °C for 1000 h. The size of the outer oxide particles increased, and the chromium content of the inner oxides decreased under tensile stress. Electrochemical measurements revealed that the charge carrier density increased, and the charge transfer resistance and film resistance were reduced under the tensile stress condition. These changes in the oxide properties are attributed to the formation of short diffusion paths such as line and surface defects due to tensile deformation.

## 1. Introduction

Nickel-based Alloy 600 materials have been used extensively to fabricate the major components of pressurized water reactors (PWRs) such as steam generator (SG) tubes, control rod drive mechanisms, and instrument penetrations. However, due to the susceptibility of such materials to corrosion damage [1,2,3], in particular, stress corrosion cracking (SCC) in both primary and secondary water environments of PWRs, Alloy 690 materials containing higher chromium content are now specified for new plants and for replacement components of operating plants.

SCC is the most critical among various modes of damage that occur in SGs. This is because cracks in SG tubes may cause an abrupt tube rupture accident, resulting in a radioactive coolant leak from the primary to the secondary system. SG tubes are subjected to various types of residual and operational stresses: The primary-to-secondary differential pressure across a tube wall during normal operation of PWRs is in the range of 8.6 to 11 MPa; thus, tensile hoop and axial stresses are induced on the secondary side of the tube by the pressure difference [4,5,6]. Heat flux from the hotter primary side to the colder secondary side also causes compressive hoop stress on the inner surface and tensile hoop stress on the outer surface [7,8,9,10]. Most of all, SG tubes suffer from mechanical stresses developed by plastic deformation such as U-bending [11,12,13,14], tube expansion at the top of the tubesheet [14,15,16,17], and denting corrosion [1,14,18,19].

SCC occurring on the secondary side of SG tube materials has been reported to be accelerated under tensile stresses in both laboratory tests and field experiences [1,2,3]. As a result, most studies have focused attention on the various factors affecting cracking under tensile stress conditions, such as microstructure of the materials [20,21,22,23,24,25,26,27], water chemistry [28,29,30,31,32,33], temperature [34,35,36], and stress and strain levels [36,37,38,39,40,41]. Furthermore, all the laboratory studies were performed in static autoclaves containing highly concentrated impurities in the test solutions and, thus, had a limited ability to simulate water chemistry conditions analogous to those of operating PWR SGs. Therefore, it is imperative to explore the role of tensile stress to determine how it affects oxide properties and corrosion processes in simulated SG water environments, compared with stress-free conditions. With this background, this work focused on the compositional and electrochemical property of oxide films grown on a nickel-based Alloy 600 material under two different stress conditions (stress-free and tensile stress) in simulated PWR secondary water at 340 °C.

## 2. Experimental Methods

### 2.1. Test Material

Nickel-based Alloy 600 was melted in a vacuum furnace, hot-rolled in the temperature range of 1050–1150 °C, and cold-rolled to a thickness of approximately 1.5 mm. The plates were solutionized at 1060 °C for 2.5 min, followed by quenching in water. The chemical composition of the alloy is given in Table 1**.** All the specimens used in this work were prepared from the annealed plates.

### 2.2. Immersion Test for Oxide Formation

**Figure 1:**
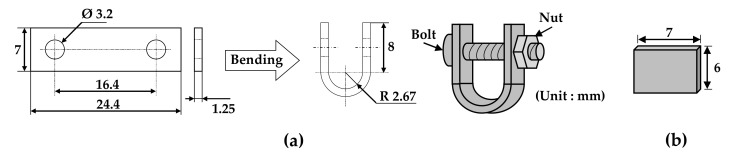
Dimensions of (**a**) a U-bend specimen and (**b**) a flat specimen for the immersion corrosion test.

Specimens with two types of stresses (tensile and stress-free) were needed to investigate the effect of tensile stress on the morphologies and compositions of the oxides. Figure 1 shows the dimensions of a U-bend specimen and a flat specimen. Each specimen was abraded with SiC paper down to #1000 grit and ultrasonically cleaned in acetone and distilled water for more than 5 min each to remove impurities. The rectangular plate with two holes was bent using a U-bend jig according to the ASTM G30-97 guideline [42]. The U-bend was assembled to maintain stress with a bolt and a nut made of Alloy 600 material in the jig without relaxation of the tensile elastic strain. As a result, tensile stress developed on the outer surfaces of the U-bend specimen. The total engineering strain (ε) on the outside of the bend was calculated to be 0.23 (elongated by 23% of its original length) using the equation ε = *T/2R* (*T*: specimen thickness and *R*: radius of the bend curvature) [42]. The strain is expressed hereinafter as a percent basis for convenience. The flat specimen represents a stress-free condition. In this manner, specimens with two types of stresses were prepared for analyses of the morphologies and compositions of oxides after the immersion corrosion test.

**Figure 2:**
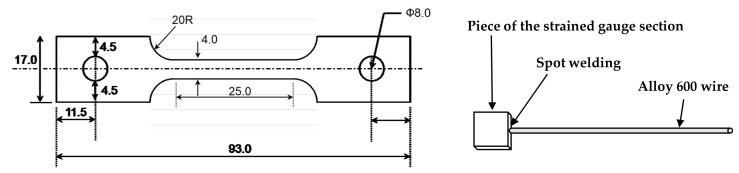
Dimensions and preparations of the tensile-strained specimens for the electrochemical tests.

To evaluate the effect of tensile stress on the electrochemical properties of the oxide layers, specimens with 30% tensile engineering strain were prepared by tensile testing of specimens with a gauge length and width of 25 mm and 4 mm, respectively, in air to the preset engineering strain level. The strained gauge sections were cut to have a surface area of approximately 1 cm^2^ along the gauge length. Each specimen was spot-welded to an Alloy 600 lead wire. These specimens were used as a working electrode for electrochemical measurements after immersion corrosion tests or for polarization tests as they were. The dimensions and preparations of the specimens are shown in Figure 2.

In general, U-shaped heat transfer tubes with various bend radii are installed in the recirculating SGs of PWRs. The plastic strains computed at a bend radius of 57 mm close to a minimum of 76 mm in the classic SGs were approximately 18% at the extrados and 35% at the intrados [43]. For this reason, the amount of 30% strain was selected, which was also similar to that on the outside of the above U-bend specimens. In this work, the U-bend specimens represent the U-bent SG tubes. In addition, the pre-strained specimens were used to simulate the strained outer surfaces of U-bent tubes and expanded tubes in a PWR SG. Meanwhile, recirculating SG tubes are installed in a PWR SG through U-bending and tube expansion methods during the SG manufacturing process. The pre-stressed (strained) tubes are then exposed to the operating secondary water chemistry of the SG without further strain. However, a specimen is continuously deformed at a strain rate during slow strain rate testing. Therefore, testing using pre-strained specimens is much more appropriate for simulating the corrosion behavior of the U-bent tubes and expanded tubes in SGs.

**Figure 3:**
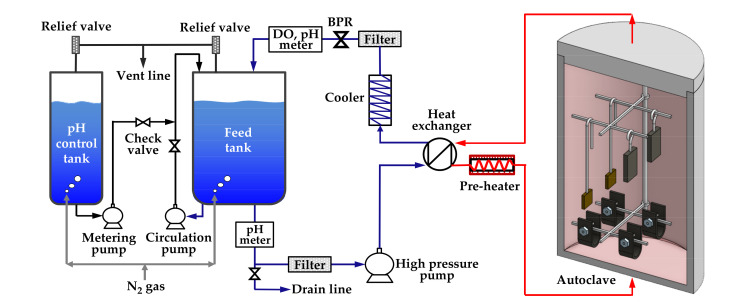
Schematic of the circulating water loop system used for the immersion corrosion test.

The immersion corrosion test was performed to form oxides on specimens with two types of stresses (stress-free and tensile stress) and on specimens with two tensile strain levels (0% and 30%) in simulated secondary water of a PWR. Figure 3 schematically shows the water-circulating system used for the test. The test solution was fed into a 316 stainless-steel autoclave by a high-pressure pump via a preheater, and the effluent was cooled-down and returned to a solution tank via a cooler, a dissolved oxygen (DO) meter, and a pH meter. Specimens were placed on specimen holders made of Alloy 600 to avoid galvanic corrosion effect in the autoclave. The test solution was prepared by adjusting the pH of deionized water to 9.0 at 25 °C with ethanolamine, which is a typical pH control agent for secondary water of PWRs. The dissolved oxygen level was controlled below 5 µg/L by continuously blowing high-purity nitrogen gas (99.999%) into the feed tank at a flow rate of 50 cm^3^/min. The temperature of flowing water adjacent to the corrosion specimens was consistently maintained at 340 °C, and the system pressure was controlled at 170 bars using a back pressure regulator (BPR). The duration of the test was 1000 h.

### 2.3. Characterization of Oxide Films

After the immersion corrosion test, the surface morphology of the oxide films formed on the specimens was observed using scanning electron microscopy (SEM: JEOL, Tokyo, Japan). Transmission electron microscopy (TEM) specimens were prepared by milling the oxidized surfaces vertically using a focused ion beam (FIB) at an acceleration voltage of 30 kV. The elemental distribution images and elemental line profiles of the oxides were obtained using energy dispersive X-ray spectroscopy (EDS) equipped on a TEM (FEI, Hillsboro, OR, USA). The acceleration voltage and resolution were 200 kV and 136 eV, respectively.

### 2.4. Electrochemical Measurements

All electrochemical experiments were conducted using a potentiostat and a three-electrode cell. A saturated calomel electrode (SCE) and a coiled platinum wire were used as the reference and counter electrode, respectively. Each test solution was deaerated by blowing high-purity nitrogen gas at a flow rate of 100 cm^3^/min before and during the test.

Electrochemical impedance spectroscopy (EIS) measurements were performed using oxidized specimens with different amounts of tensile strain (0% and 30%) to investigate the electrochemical property of oxide layers grown in simulated PWR secondary water at 340 °C. The EIS test solution was a borate solution (0.05 M H_3_BO_3_ + 0.075 M Na_2_B_4_O_7_·H_2_O) with pH 9.2 at 25 °C. After the open-circuit potential (OCP) was stabilized, EIS measurements were conducted at the OCP using an AC amplitude of ±10 mV and a frequency range from 10 mHz to 100 kHz.

Capacitance responses of the oxide films formed in simulated PWR secondary water at 340 °C were measured in a borated buffer solution (0.05 M H_3_BO_3_ + 0.075 M Na_2_B_4_O_7_) at 25 °C. When the OCP stabilized, capacitance values were measured at a frequency of 1000 Hz in a potential range from +1.0 V to −1.5 V. An AC signal with a 10 mV amplitude was applied to the cell.

Potentiodynamic polarization tests were performed using fresh specimens with different strains (0% and 30%) that were not exposed to the immersion corrosion condition. The solution for the polarization tests had the same solution chemistry as that used in the immersion test. The solution temperature was maintained at 80 °C using a heating mantle. Cathodic preconditioning was done at −0.5 V versus the OCP for 3 min after the potential reached the stable OCP. After that, polarization scans were run from −0.4 V to +1.6 V versus the OCP at a scan rate of 0.5 mV/s. The corrosion current densities (i_corr_) of the test materials were calculated using the Tafel extrapolation method from cathodic polarization curves.

## 3. Results

### 3.1. Morphology and Chemical Composition of Oxide Films

**Figure 4:**
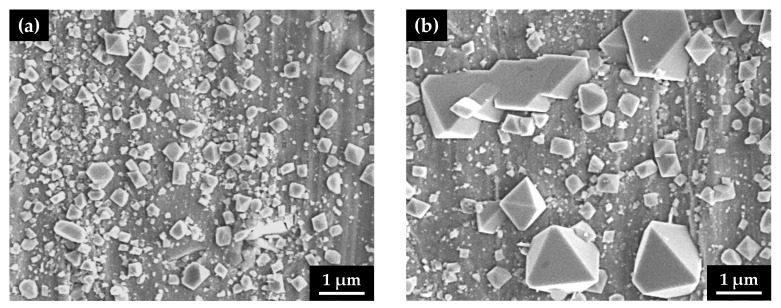
SEM micrographs of the oxidized surfaces of Alloy 600 specimens with two different stress states in simulated PWR secondary water: (**a**) stress-free specimen and (**b**) tensile-stressed specimen.

After the immersion corrosion test, the surface morphology of the oxide films was observed using SEM on the outer surfaces of the U-bend specimens along the apex line, corresponding to a maximum tension region. Oxide films grown on the flat specimens without stress were also examined using SEM. As shown in Figure 4, numerous small, polyhedral particles were formed on the surfaces, regardless of the stress type. However, the size of the surface particles on the specimen with the tensile stress markedly increased to approximately 2 μm, indicating that the corrosion from the alloy substrate was significantly accelerated by the tensile stress.

**Figure 5:**
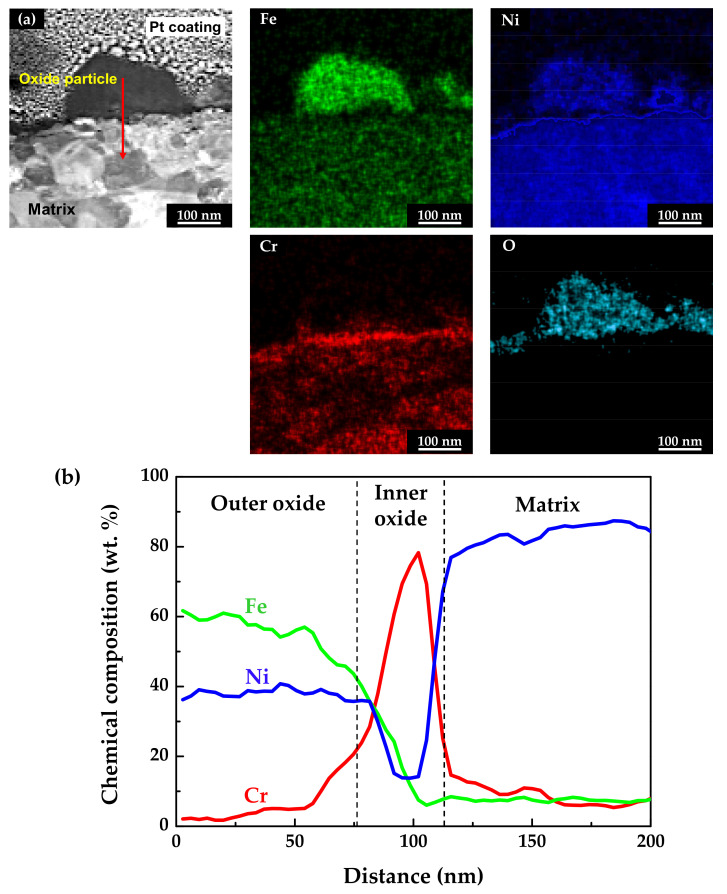
STEM image and EDS analyses of oxide films formed on Alloy 600 specimens under the stress-free condition: (**a**) EDS elemental maps and (**b**) EDS elemental line profiles.

The chemical compositions of the oxidized specimens were analyzed using scanning TEM (STEM) and EDS. TEM foils were prepared from the cross-sections of the specimens using the FIB technique. Figure 5 shows the STEM micrographs and EDS analysis results of the stress-free specimen. As shown in Figure 5a, the EDS elemental mapping images revealed that the outer particles were oxides composed mainly of iron and nickel, with a minor amount of chromium. In addition, a chromium-enriched, thin oxide layer was observed not only on the surface adjacent to the matrix, but also beneath the oxide particles. The chemical compositions of the above oxide layers were measured using EDS line profiling along the red arrow direction denoted in the STEM image. The obtained results were then normalized to obtain the weight percent of the major alloy elements (nickel, chromium, and iron). As shown in Figure 5b, the outer oxide particles were determined to have a chemical composition similar to nickel ferrite, NiFe_2_O_4_. The EDS line profiles also clearly revealed that the chromium-enriched inner layer was formed beneath the outer oxide particles. Chromium up to approximately 80 wt.% was detected in the inner layer. Note that the chromium content of the matrix was 15 wt.%, as shown in Table 1. In summary, the oxide films consisted of double layers with an iron- and nickel-rich outer layer and a chromium-rich inner layer. Therefore, the chemical compositions of the oxide layers indicate that the chromium-rich inner layer was formed through preferential dissolution of iron and nickel, and that the dissolved iron and nickel ions were precipitated to form the iron- and nickel-rich oxide particles on the outer surface.

**Figure 6:**
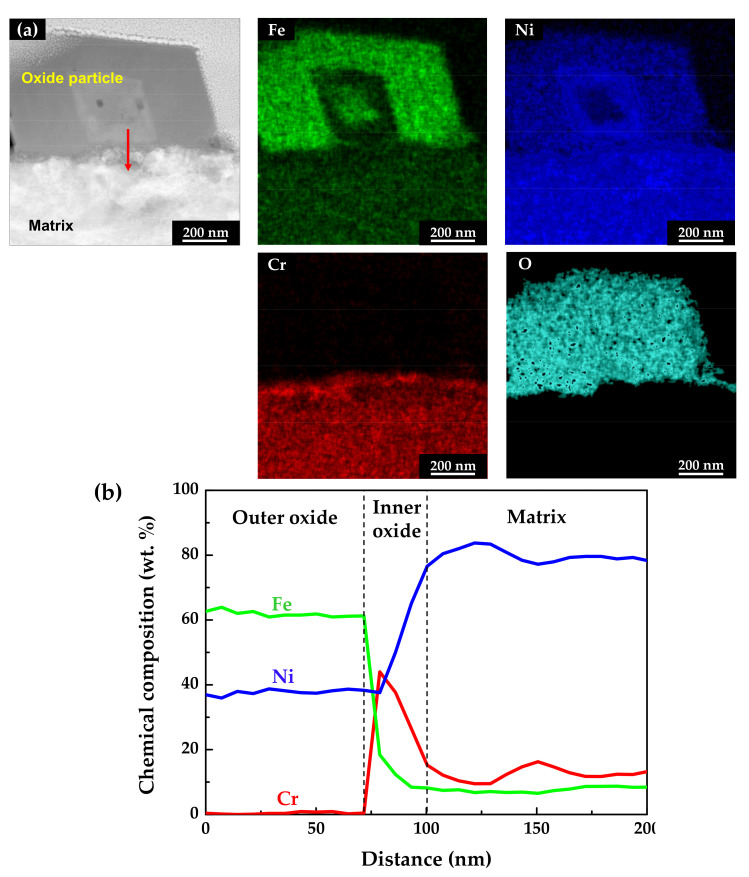
STEM image and EDS analyses of oxides formed on Alloy 600 specimens under the tensile stress condition: (**a**) EDS elemental maps and (**b**) EDS elemental line profiles.

Figure 6 shows the STEM micrographs and EDS results of the specimen oxidized under the tensile stress condition. The EDS maps and line profiles revealed that the oxide films had a double-layered structure and that the outer particles were oxides with the composition of NiFe_2_O_4_, similar to those of the stress-free specimen. The chromium-enriched inner oxide layer was also clearly found. However, the chromium content in the inner layer was around 40 wt.%, only half of that detected in the stress-free specimen. This indicates that the dissolution rate of chromium was relatively accelerated compared to that under the stress-free condition, although iron and nickel were still preferentially dissolved.

### 3.2. Polarization Behavior of Tensile-Strained Specimens

**Figure 7:**
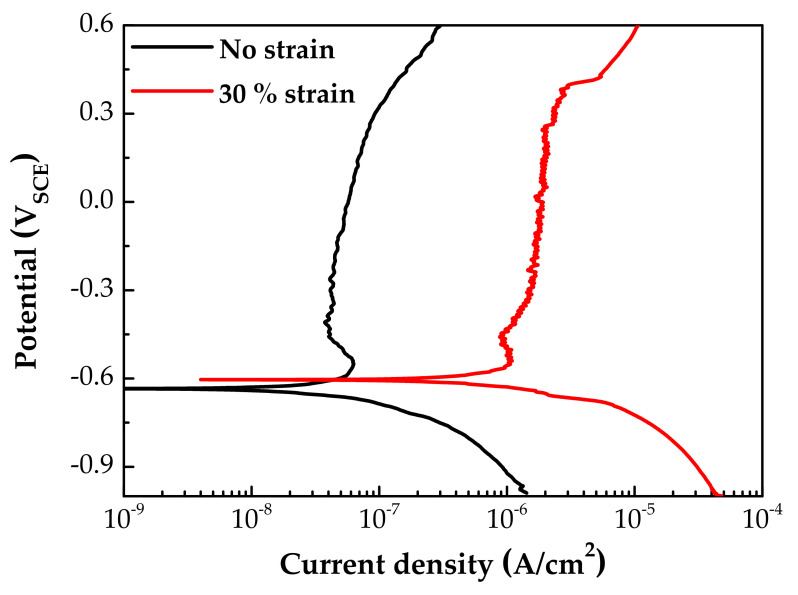
Potentiodynamic polarization curves of fresh Alloy 600 specimens with and without 30% tensile strain.

Figure 7 shows the potentiodynamic polarization curves of specimens with and without 30% tensile strain. The corrosion potentials (E_corr_) were little affected by the strain. However, both the anodic and the cathodic polarization current densities of the strained specimen were significantly increased by the plastic tensile strain. In particular, the corrosion current density at E_corr_ and anodic current densities above the E_corr_ were 20–30 times greater than those of the stress-free specimen. It is believed that the above changes in particle size and chemical composition of oxides, as well as polarization current, are closely related to the electrochemical property of the oxides. Therefore, the EIS and capacitance behavior of oxide films were analyzed, as described below.

### 3.3. EIS Analyses of Oxide Films

**Figure 8:**
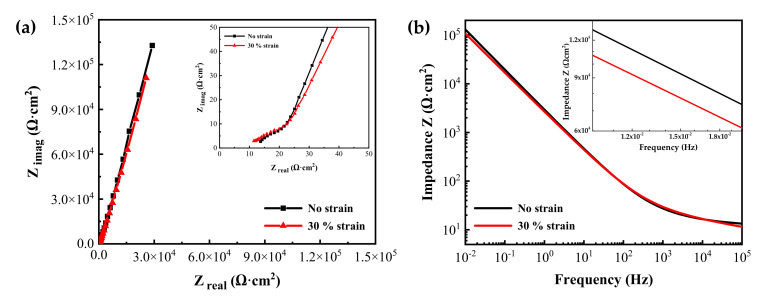
(**a**) Nyquist and (**b**) Bode plots of oxide films grown on Alloy 600 specimens with and without 30% tensile strain in simulated PWR secondary water at 340 °C.

Figure 8 shows the EIS measurement results of the oxide films formed on specimens with two different tensile strain levels in simulated PWR secondary water at 340 °C for 1000 h. The Nyquist plots revealed one capacitive arc in the high-frequency region and a long tail in the low-frequency region. The impedances from the 30% strained specimen were smaller than those from the stress-free specimen at lower frequencies. The Bode plots also showed that the impedance values of the strained specimen were slightly smaller than those of the stress-free specimen at low frequencies. The impedance at low frequencies is related to the charge transfer in the Faradaic corrosion process [44,45,46,47]. Therefore, the EIS result indicates that the oxide film on the tensile-stressed specimen has a relatively lower corrosion resistance.

**Figure 9:**
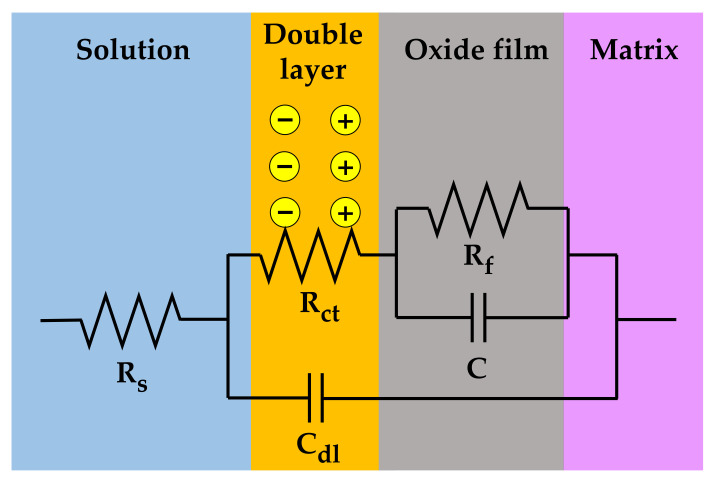
Electrical equivalent circuit used for the EIS data analysis of the oxide films grown on Alloy 600 specimens in simulated PWR secondary water at 340 °C.

The measured EIS data were analyzed in more detail by fitting them to the electrical equivalent circuit shown in Figure 9, where R_s_ is the resistance of the test solution, R_ct_ is the charge transfer resistance of the Faraday processes, C_dl_ is the capacitance of the electric double layer, R_f_ is the resistance of the oxide films, and C is the capacitance of the oxide films. The circuit model was constructed to simulate the electrochemical impedance behavior at the solution/oxide film interface and inside the oxide films. The EIS data were fitted using the Gamry Electrochem Analyst software. The electrochemical impedance parameters obtained for the equivalent circuit are listed in Table 2. The charge transfer resistance and film resistance decreased by approximately 20% and 30%, respectively, when the oxide films were grown on the 30% strained specimen. During the corrosion process, metal cations migrate toward the solution through defects in the oxide films and diffuse into the solution, while oxygen anions move from the solution toward the matrix. Therefore, this result indicates that the charge transfer reactions at the film/solution interface occurred more actively on the strained specimen than on the stress-free specimen. This is consistent with the oxide particle distribution (Figure 4) and the polarization behavior (Figure 7).

### 3.4. Capacitance Behavior of Oxide Films

The space charge capacitance of an oxide film/solution interface can be described by the following equation according to the Mott–Schottky theory [48,49,50,51,52,53,54,55].

(1) $$\frac{1}{C^{2}}=\frac{2}{{\varepsilon \varepsilon }_{0}{\mathrm{qN}}_{c}}\left(E-E_{\mathrm{fb}}-\frac{\mathrm{kT}}{q}\right)$$

where C is the film capacitance, ε is the dielectric film constant, ε_0_ is the vacuum permittivity (8.854 × 10^−14^ F·cm^−1^), q is the elementary electric charge (1.602 × 10^−19^ C), N_c_ is the charge carrier density, E is the applied potential, E_fb_ is the flat band potential, k is the Boltzmann constant (1.38 × 10^−23^ J·K^−1^), and T is the absolute temperature. Therefore, the charge carrier density in the film can be calculated from the slope (2/εε_0_qN_c_) of a Mott–Schottky plot.

**Figure 10:**
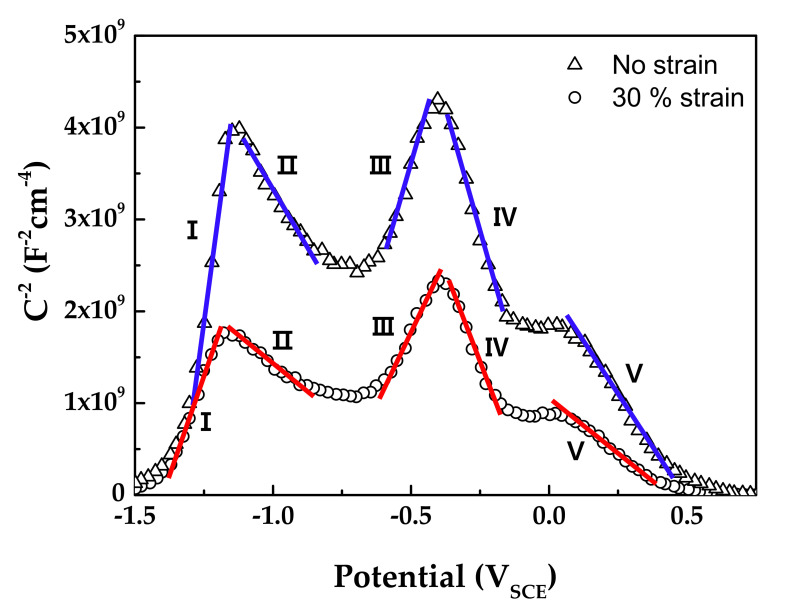
Mott–Schottky plots of oxide films formed on Alloy 600 specimens in simulated PWR secondary water at 340 °C.

Figure 10 shows the Mott–Schottky plots of oxide films formed on Alloy 600 specimens in simulated PWR secondary water at 340 °C. The capacitance values of films formed on the strained specimen were larger than those on the stress-free specimen. Because capacitance and impedance are in reverse proportion to each other, this result indicates that the film resistance of the strained specimen was smaller than that of the stress-free specimen, in agreement with the EIS data. The Mott–Schottky plots revealed that the oxide films were composed of both n-type and p-type semiconductors. Here, the positive slopes in the regions I and III represent an n-type semiconductor response, while the negative slopes in the regions II, IV, and V represent a p-type semiconductor response. Iron oxides behave as an n-type semiconductor and chromium oxides behave as a p-type semiconductor [55,56,57,58]. Therefore, the above capacitance responses reflected the double-layered oxide films with the outer ferrite-rich layer and the chromium-rich inner layer observed in Figure 5 and Figure 6. The calculated charge carrier densities are listed in Table 3. The dielectric constant of the film was assumed to be 12 [59,60]. The total charge carrier (donor for an n-type and acceptor for a p-type) densities in the oxide films of the 30% strained specimen were two times greater than those in the films of the stress-free specimen. This demonstrates that the point defects in the oxide films increased significantly when the oxide films were grown on the strained surface. The decrease in the film resistance (Table 2) can be attributed to the formation of the defective oxide films.

## 4. Discussion

When comparing the stressed with the stress-free condition, important observations obtained under the tensile condition can be summarized as follows: the size of the outer oxide particles increased remarkably (Figure 4), the chromium content in the inner oxide layer was halved (Figure 5 and Figure 6), the anodic polarization current density increased significantly (Figure 7), the EIS resistance of the film was decreased (Table 2), and the defect density in the film was doubled (Table 3). Therefore, it is crucial to examine thoroughly how they were interconnected in the corrosion processes.

It has been reported that double-layered oxide films with a nickel ferrite outer layer and a chromium-enriched inner oxide layer are formed on nickel-based alloys and stainless steels in lithiated and borated primary water of PWRs [54,61,62,63,64]. As shown in Figure 4, Figure 5 and Figure 6, oxide films with similar morphology and composition were observed, indicating that oxide films with a double-layered structure are also grown on Alloy 600 even in the simulated secondary water of PWRs. When considering that the outer oxide particles are grown by precipitation of metal cations dissolved from the alloy matrix [64,65,66], the drastic increase in the size of the outer oxide particles in Figure 4 indicates that the anodic dissolution and release of the alloy elements was accelerated under the tensile stress condition. This feature is consistent with the electrochemical measurement showing that the anodic dissolution current density increased approximately 20–30-fold (Figure 7) on the tensile-strained specimen. The dissolved iron and nickel cations were the source of the outer nickel ferrite particles; thus, the particle size increased as the dissolved metal cations increased. Therefore, the polarization behavior provides the electrochemical basis for why the oxide particle distribution was affected by the tensile stress.

Meanwhile, chromium-rich inner layers are known to be formed through solid-state diffusion of alloy elements and oxygen anions [66,67]. Therefore, the corrosion resistance of alloys is strongly dependent on the composition and defect density of the inner oxide layer [68]. Although a chromium-enriched inner layer was also formed under tensile stress, the chromium content in the layer was at most half compared to that formed under the stress-free condition (Figure 5 and Figure 6), resulting in deterioration of the protectiveness against corrosion. It is, therefore, apparent that the fast corrosion behavior of the tensile-stressed specimens can be attributed to the lowered chromium content in the inner layer. As a result, charge transfer reactions of metal cations and oxygen anions through the oxide films were enhanced. As alloy elements dissolved fast in the solution, as shown in Figure 7, more electrons entered the alloy and more metal cations diffused into the solution, thereby increasing the kinetics of charge transfer. It is evident from the EIS results that the charge transfer resistance and film resistance declined under the tensile stress condition. Metal cations and oxygen anions migrate through point defects in oxide films [69,70]. Therefore, the increase in defect density in the strained films resulted in a decrease in the charge transfer and film resistance.

**Figure 11:**
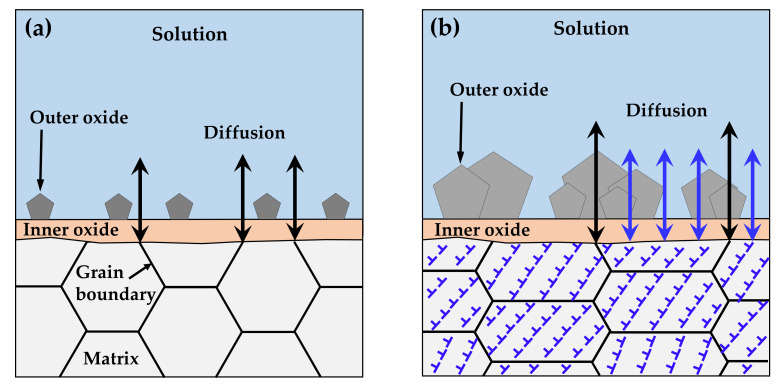
Accelerated corrosion of tensile-deformed Alloy 600 due to generation of diffusion paths: (**a**) before and (**b**) after deformation.

It is important to discuss the reasons for the increased corrosion and for the decrease in the chromium content of the inner layer under tensile stress. As described in Section 3, the chromium-enriched inner oxide layer resulted from the preferential dissolution of iron and nickel in the matrix. The diffusion of alloy elements and oxygen ions occurred predominantly along grain boundaries with numerous defects due to crystal misorientation, as opposed to through the ordered lattices, as shown in Figure 11a. According to Robertson’s study [67], the diffusion of iron and nickel cations occurs much faster than for chromium cations. Therefore, iron and nickel cations would reach the matrix/solution interface quickly, while chromium would be oxidized in the inner layer, resulting in the formation of the nickel ferrite outer layer and the chromium-rich inner layer. However, numerous line defects such as dislocations and surface defects due to the clustering of line defects develop over specimens by tensile deformation [71]. These defects provide short paths for diffusion and, thus, charge transfer reactions are increased, as observed in this work. Consequently, the diffusion of ions is no longer limited to grain boundaries and occurs over the whole surface of the deformed crystal, as depicted in Figure 11b. As a result, the diffusion of chromium would be enhanced, resulting in a decrease in the chromium content of the inner layer under tensile stress. Similarly, a significant increase in the depth of oxidation penetration was found in the highly stressed regions of a nickel-based RR1000 alloy that was exposed to fatigue loading at 750 °C in air [72], indicating that oxygen diffusion was enhanced by tensile stress. In addition, rapid dissolution of the alloy elements under tensile stress would result in the formation of defective oxide films. The increase in the defect density and the decrease in the film resistance may reflect this feature of the oxide films.

## 5. Conclusions

(1) Oxide films with an iron- and nickel-rich outer layer and a chromium-rich inner layer were grown on Alloy 600 specimens regardless of tensile stress. However, the particle size on the outer layer increased and the chromium concentration in the inner layer decreased on the tensile-stressed specimens, indicating that the anodic dissolution of the alloy elements including chromium was accelerated. This was evidenced by the drastic increase in the polarization current density under the tensile stress condition.
(2) The charge carrier density increased, and the charge transfer resistance and film resistance were reduced when the oxide films were formed on the tensile-strained specimens, indicating that the diffusion of metal elements and oxygen through the films was expedited. These results are attributed to the generation of short diffusion paths such as line and surface defects due to tensile deformation.
(3) The morphology, composition, and electrochemical properties of the oxide films were closely interconnected and were affected strongly by the tensile stress. According to the results obtained in this work, the susceptibility to SCC of Alloy 600 under tensile stress conditions in secondary water of PWRs can be attributed to the increased dissolution of alloy elements and the resulting film formation with less chromium content and more point defect density.

## Figures and Tables

**Table 1:** Chemical composition of Alloy 600 (wt.%).

Ni	Cr	Fe	C	Si	Mn	Ti	Al	S	N	Cu	Co
76.01	15.20	7.82	0.023	0.26	0.21	0.25	0.17	<0.001	0.003	0.034	0.020

**Table 2:** Electrochemical impedance parameters for the oxide films grown on Alloy 600 specimens in simulated PWR secondary water at 340 °C.

Specimen	R_s_ (Ω·cm^2^)	R_ct_ (Ω·cm^2^)	C_dl_ (10^−5^ F·cm^−2^)	R_f_ (10^5^ Ω·cm^2^)	C (10^−5^ F·cm^−2^)
No strain	9.4	1117	2.08	3.94	6.15
30% strained	12.3	911	2.21	2.75	7.63

**Table 3:** Donor (N_d_) and acceptor (N_a_) densities in oxide films calculated from the Mott–Schottky analyses.

Specimen	Region I	Region II	Region III	Region IV	Region V
	(N_d_, ×10^21^ cm^−3^)	(N_a_, ×10^21^ cm^−3^)	(N_d_, ×10^21^ cm^−3^)	(N_a_, ×10^21^ cm^−3^)	(N_a_, ×10^21^ cm^−3^)
No strain	0.40	2.42	1.40	1.17	2.58
30% strained	1.59	5.04	2.26	1.80	5.15

## Data Availability

Not applicable.
